# A Stoichioproteomic Analysis of Samples from the Human Microbiome Project

**DOI:** 10.3389/fmicb.2017.01119

**Published:** 2017-07-18

**Authors:** Briana Vecchio-Pagan, Sharon Bewick, Kumar Mainali, David K. Karig, William F. Fagan

**Affiliations:** ^1^Research and Exploratory Development Department, Johns Hopkins Applied Physics Laboratory, Laurel MD, United States; ^2^Department of Biology, University of Maryland, College Park MD, United States

**Keywords:** human microbiome project (HMP), stoichioproteomics, gut, oral, skin, vaginal, nasal, C:N ratio

## Abstract

Ecological stoichiometry (ES) uses organism-specific elemental content to explain differences in species life histories, species interactions, community organization, environmental constraints and even ecosystem function. Although ES has been successfully applied to a range of different organisms, most emphasis on microbial ecological stoichiometry focuses on lake, ocean, and soil communities. With the recent advances in human microbiome research, however, large amounts of data are being generated that describe differences in community composition across body sites and individuals. We suggest that ES may provide a framework for beginning to understand the structure, organization, and function of human microbial communities, including why certain organisms exist at certain locations, and how they interact with both the other microbes in their environment and their human host. As a first step, we undertake a stoichioproteomic analysis of microbial communities from different body sites. Specifically, we compare and contrast the elemental composition of microbial protein samples using annotated sequencing data from 690 gut, vaginal, oral, nares, and skin samples currently available through the Human Microbiome Project. Our results suggest significant differences in both the median and variance of the carbon, oxygen, nitrogen, and sulfur contents of microbial protein samples from different locations. For example, whereas proteins from vaginal sites are high in carbon, proteins from skin and nasal sites are high in nitrogen and oxygen. Meanwhile, proteins from stool (the gut) are particularly high in sulfur content. We interpret these differences in terms of the local environments at different human body sites, including atmospheric exposure and food intake rates.

## Introduction

The study of ecological stoichiometry (Sterner and Elser, 2002) derives from observed commonalities and differences in the elemental compositions of organisms (Elser et al., 1996), populations and communities (Moe et al., 2005). That is, whereas all life is primarily composed of carbon (C), hydrogen (H), oxygen (O), nitrogen (N), phosphorus (P), and sulfur (S), there is nonetheless large variation in the ratios of these elements across different species (Andersen and Hessen, 1991; Hessen and Lyche, 1991; Elser et al., 1996) and systems (Cebrian and Kingsolver, 1999; Elser et al., 2000a). Such variation is important because it provides valuable biological understanding into both the nature of the organisms in a particular ecosystem (Elser et al., 2000a,c; Fagan et al., 2002; Denno and Fagan, 2003), and the environmental conditions that these organisms face (Cebrian and Kingsolver, 1999; Elser et al., 2000a). Of particular note, because all organisms require C, H, O, N, P, and S, biomass ratios of these elements can provide insight into which are limiting (i.e., prevent further growth) for specific types of taxa or for taxa from specific regions.

Much of the work in the field of ecological stoichiometry has focused on C:N:P ratios (Elser et al., 2000b,c, 2003). Species growth rate, for example, has been associated with an organism’s absolute C:N ratio, while C:N ratio variances have been linked to both growth rate and trophic guild (Rhee, 1978; Urabe, 1993; DeMott et al., 1998; Elser et al., 2000a,c; Denno and Fagan, 2003; Lemoine et al., 2014). Broad trends in organismal elemental composition have also been documented as a function of ecosystem type. Marine autotrophs, for instance, are more nutrient rich (lower C:P and C:N ratios) than freshwater autotrophs, which are more nutrient rich than terrestrial autotrophs (Elser and Hassett, 1994; Elser et al., 2000a; Sterner and Elser, 2002), while marine autotrophs exhibit smaller ranges in elemental ratios as compared to autotrophs from terrestrial systems (Elser et al., 2000a, 2006).

As primary nutrient remineralizers (Barsdate et al., 1974; Goldman et al., 1987; Chrzanowski and Kyle, 1996), and some of the most N- and P-rich of all living things (Sterner and Elser, 2002), the ecological stoichiometry of bacteria is particularly interesting. Some of the first studies of bacterial stoichiometry focused on the elemental composition of the bacterial cell (Herbert, 1961, 1976; Herbert et al., 1971). More recently, however, the emphasis has shifted to environment driven differences. Tezuka (1990), for example, studied the relationship between C:N ratios in culture medium and in bacterial biomass for an assemblage of lake bacteria. His study, and others (Nakano, 1994; Chrzanowski and Kyle, 1996) showed that there is a positive correlation between nutrient supply rates and bacterial biomass in microbial communities, and that this relationship is largely driven by shifts in the relative ratios of different bacterial populations (Makino et al., 2003).

Although understudied relative to classic ecology systems (e.g., oceans, lakes, and soils), it is reasonable to hypothesize that the relative abundances of bacteria across different human microbiomes (Turnbaugh et al., 2007; Peterson et al., 2009; Proctor, 2011; Gevers et al., 2012; Human Microbiome Project Consortium, 2012a) might be similarly governed by nutrient supply rates. Naively, one might expect that all human microbiomes should be relatively nutrient rich. This is because the human tissues and byproducts that provide the substrates for these microbes should be nutrient rich by virtue of the human lifestyle and trophic guild (Fagan et al., 2002). However, while human microbiomes may be overall more nutrient rich than those from plants or lakes or other nutrient-poor environments, differing nutrient supply ratios across body sites could still lead to different nutrients being limiting in one region relative to another. Nutrient availability in the gut, for instance, is at least partially a function of food intake, whereas nutrient availability on the skin depends primarily on host tissue and host produced compounds (Belkaid and Segre, 2014). Indeed, some body sites are typically thought of as providing more or less nutrients, with the gut being traditionally considered a nutrient-rich site, and the skin being considered a nutrient-poor site (Belkaid and Segre, 2014; Belkaid and Tamoutounour, 2016). It is already well known that bacterial abundances vary strongly among body sites (Human Microbiome Project Consortium, 2012a). If this is at least partially a result of differing nutrient limitations, then the elemental compositions of microbial populations should vary from one body site to the next. As an example, body sites with large aerobic niches (e.g., skin and nares) might be expected to contain microbial biomass with a higher oxygen content (Acquisti et al., 2007). By contrast, the anaerobic vagina contains nitrogen-rich urea and protein secretions (Geshnizgani and Onderdonk, 1992), and thus might be expected to exhibit microbial biomass with high nitrogen ratios.

As a first step toward testing the hypothesis that variation in human microbiomes reflects underlying differences in nutrient availability, we consider the elemental compositions of microbial proteins from different body sites. That is, we take a stoichioproteomic approach (Baudouin-Cornu et al., 2001; Elser et al., 2006, 2011) wherein we analyze the C, N, O, and S content of microbial proteins among gut, oral, nasal, vaginal and skin microbiome samples. Previous work has shown that even single atom changes in the amino acid (AA) composition of microbial proteins can have large fitness consequences under nutrient-limiting conditions (Bragg and Wagner, 2009), suggesting that variation in microbial protein composition across body sites could reflect differing selective forces resulting from varying nutrient supplies. Further supporting this claim, organisms suffering chronic deficiency in certain nutrients (e.g., C, N or S) are known to preferentially substitute AAs that are low in the limiting nutrients. For example, S and C assimilatory pathways in both *Escherichia coli* and *Saccharomyces cerevisiae* show evidence for depletion of S and C, respectively (Baudouin-Cornu et al., 2001). Likewise, under conditions of sulfur limitation, certain cyanobacteria express sulfur-depleted versions of abundant proteins (Mazel and Marliére, 1989). Thus, our expectation is that if there are differences in C, N, S or O limitation across different human body sites, then we should see body site differences in the content of microbial proteins as well.

Unfortunately, whereas a lack of body site variation in protein elemental composition would provide strong evidence that nutrient limitation does not differ across body sites, the inverse is not necessarily true – i.e., differences in protein composition do not necessarily imply differences in nutrient supply. One reason for this is that protein elemental composition is known to depend on the GC content of the underlying coding sequences (CDSs). The GC content of CDSs, however, is under different selective forces (Rocha and Danchin, 2002; Musto et al., 2006) that are completely distinct from protein composition and, by extension, nutrient limitation. Thus, body site differences in protein composition could indicate differing selective forces on GC content, rather than protein composition *per se*. For similar reasons, chemical and/or structural constraints on protein shape and function might also select for body site differences in elemental composition. Acidic sequences, for instance, are higher in oxygen content, while basic AAs are higher in nitrogen content. Requirements for these functional groups and others at certain body sites may thus bias element usage, independent of nutrient supply.

Interestingly, we do find significant body site differences in the C, N, S, and O content of microbial proteins. Supporting the hypothesis that this is a result of selection acting at the level of proteins, rather than GC content, we find that body site differences in protein composition remain significant, even after accounting for the dependencies of protein composition on GC content. Further supporting our hypothesis that body site differences are a result of nutrient limitation, we find that AAs with similar functional groups are often used differentially at different body sites based on specific AA element ratios. Although it is impossible to rule out other forces of selection that might indirectly select for differences in element composition across body sites (e.g., thermal stability requirements of proteins), our results provide strong preliminary evidence that nutrient limitation may vary across the human body and that this, in turn, may impact the associated structure of the human microbiome.

## Materials and Methods

### Input Data

The Human Microbiome Project’s (HMP) gene indices dataset was downloaded from their ftp server on 07/10/2016^1^. At the time of download, 690 samples representing 15 body sites were available. In this dataset, person identity is masked. However, based on sample counts, samples were collected from a minimum of 139 people. The HMP dataset description suggests that samples came from a much larger group (Human Microbiome Project Consortium, 2012a). That said, at least some of the samples in our dataset are likely from different body sites on the same individual. Although we cannot statistically account for this form of non-independence because of the masking of identities, we do not expect it to significantly impact our predictions, because taxonomic body site differences are known to be large relative to interpersonal variation at a single body site (Human Microbiome Project Consortium, 2012b).

Samples were sequenced at four different institutions, specifically Baylor College of Medicine, the Broad Institute, the J. Craig Venter Institute and the Washington University Genome Sequencing Center. Since HMP sample collection and processing followed a standard protocol at all institutions, we did not account for potential institution differences. Supporting this decision, analysis of a reduced sample set considering only those samples processed at Washington University Genome Sequencing Center gave similar results to our full analysis (see Appendix I, Figure A.1.1). More information on the methods used for sample collection, and specific details about sample characteristics are available from the HMP website^2^.

The HMP gene indices dataset was previously processed using HMP’s Metagenomics Prokaryotic Annotation Pipeline^3^. This yielded putative protein sequences for each of the 690 shotgun sequencing samples. These putative protein sequences were downloaded both in nucleotide and AA multi-FASTA formats.

**Table 1** shows the breakdown in sample numbers by body sites.

**Table 1:** Body sites used in study; two-letter codes for minor body sites are shown in brackets.

Major body site	Samples	Minor body site	Samples
Skin	26	Left retroauricular crease (Lr)	9
		Right retroauricular crease (Rr)	17
Stool/Gut	139	Stool (St)	139
Nasal	87	Anterior nares (Na)	87
Vagina	56	Vaginal Introitus (Vi)	3
		Mid-vaginal (Mv)	2
		Posterior fornix (Pf)	51
Oral	382	Saliva (Sa)	3
		Throat (Th)	7
		Buccal mucosa (Bm)	107
		Palatine tonsils (To)	6
		Tongue dorsum (Td)	128
		Subgingival plaque (Sb)	7
		Supragingival plaque (Sp)	118
		Attached keratinized gingiva (Ak)	6


### Protein Elemental Content

Our baseline goal was to determine differences in protein elemental composition across body sites. To this end, we used the AA sequences available for all identified CDSs in the HMP gene indices dataset. AA sequences for the CDSs in any given sample were used to infer the average protein elemental composition (C, O, N, and S) of that sample based on the known elemental compositions of each AA. Specifically:

$$X=\frac{1}{N}\Sigma_{i=1}^{N}\frac{\Sigma_{j=1}^{20}x_{j}n_{i,j}}{\Sigma_{j=1}^{20}T_{j}n_{i,j}}$$

where *X* is the average fraction of the focal element in proteins from the sample, *N* is the total number of CDSs in the sample, *n*_i__,__j_ is the number of AAs of type *j* in CDS *i*, *x*_j_ is the number of atoms of the focal element in an AA of type *j* and *T*_j_ is the total number of atoms in an AA of type *j*. For the analysis presented in the main text, we use *x*_j_ and *T*_j_ reflective of full AAs, i.e., including both the side chain and the backbone (but see Appendix I, Figures A.1.2–A.1.6 for analysis with just the side chains).

### Protein Amino Acid Content

To further explore underlying chemical constraints that might govern body site differences in protein composition, we examined AA use, focusing on AAs with N, O, and S, because these elements are present in the side-chains of less than half of common AAs. From the AA sequences provided, we determined the average AA content of a sample as follows:

$$Z_{j}=\frac{1}{N}\overset{N}{\underset{i=1}{\Sigma }}\frac{n_{i,j}}{A_{i}}$$

where *Z*_j_ is the fraction of AAs of type *j*, *n*_i__,__j_ is the number of AAs of type *j* in CDS *i*, *A*_i_ is the total number of AAs in CDS *i*, and *N* is the total number of CDSs in the sample.

### Coding Sequence GC Content

Because protein elemental composition is known to vary with GC content of the corresponding CDSs, we also examined CDS GC content. Using nucleotide sequences provided in the HMP gene indices dataset, we inferred the average GC content of a sample as follows:

$$Y=\frac{100}{N}\Sigma_{i=1}^{N}\frac{\Sigma_{j=1}^{B_{i}}1_{\text{GC}}\left(y_{i,j}\right)}{B_{i}}$$

where *Y* is the average GC content of the CDSs in the sample, *N* is the total number of CDSs in the sample, $1_{\text{GC}}\left(y\right)=\left\{ \begin{array}{c} 1y=G,C\\ 0y=A,T \end{array}\right.$ is an indicator function, *y*_i__,__j_ is the *j*th nucleotide in the *i*th CDS, and *B*_i_ is the length of the *i*th CDS (i.e., total number of nucleotides).

### Body Site Comparison

To determine whether protein elemental fractions or AA composition differed from one body site to another, we compared elemental ratios across the 15 specific body sites in **Table 1**. We also grouped these body sites into five broader categories, also shown in **Table 1**. Differences in median elemental/AA fractions among body sites were determined based on Dunn’s test, which is a pairwise multiple comparisons procedure based on rank sums. False Discovery Rate (FDR) was controlled using the Benjamini-Hochberg adjustment. Differences in elemental fraction variances among body sites were also determined. This was done using Levene’s test (only for the five major body sites). Again, FDR was controlled using the Benjamini-Hochberg adjustment. In the main text, we use boxplots to display differences in protein elemental/AA composition among body sites. Following standard convention, the center line of the boxplot is the median elemental/AA fraction (or ratio), the box defines the lower and upper quartiles of observed elemental fractions, and the ‘whiskers’ define the minimum and maximum elemental fractions, excluding outliers. Outliers are taken as any points greater (less) than 1.5× the upper (lower) quartile.

### Element versus GC Analysis

To determine whether body site differences in protein elemental composition were significant, even after accounting for body site variation in GC content, we used analysis of covariance (ANCOVA) models. Type I sums of squares was used to calculate an *F*-value and to determine the significance of GC content and major body site on each elemental fraction and the C:N ratio.

All code from our work is available at: http://www.clfs.umd.edu/biology/faganlab/ecological-stoichiometry.html.

## Results

### Carbon Content

**FIGURE 1:**
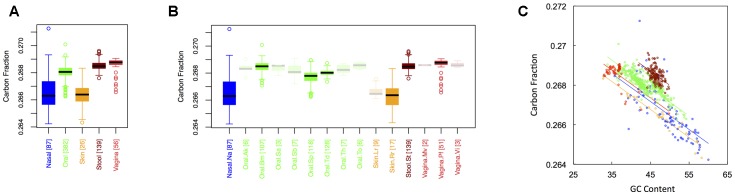
Boxplots showing the average carbon content of microbial proteins as a function of human body site for **(A)** the 5 major sites and **(B)** the 15 minor sites in our study. The number of samples from each site is indicated in square brackets on axis labels. Faded boxes are used for sites with fewer than 10 samples. Differences in carbon content were tested with Dunn’s test. Statistics are summarized in Appendix II, Tables A.2.1, A.2.2; **(C)** Scatterplot of carbon content versus GC content with associated regression lines from our ANCOVA analysis for major body sites: nasal (blue), oral (green), skin (yellow), stool (brown), and vaginal (red).

**Figure 1** shows boxplots of the average carbon fraction (see “Materials and Methods”) of microbial proteins across the 5 major (**Figure 1A**) and 15 minor (**Figure 1B**) body sites in our study. Interestingly, all major body sites differed in carbon content except for those from nasal and skin regions and those from vaginal sites and stool (see Appendix II, Table A.2.1). Carbon content was lowest at nasal and skin sites, higher at oral sites, and reached a maximum at vaginal sites and in stool (C_stool_ ∼ C_vaginal_ > C_oral_ > C_skin_ ∼ C_nasal_). Similar trends held for minor body sites (see Appendix II, Table A.2.2). For example, the nares (nasal) had lower carbon content than any minor body site except for the left and right retroauricular creases (skin). Nevertheless, analysis of minor body sites did uncover the existence of additional, fine-scale variation that was not obvious from the five major body sites. Most notably, in the oral microbiome, there were significant differences in the carbon content of proteins from the buccal mucosa as compared to those from both supragingival plaque and the tongue. Likewise, proteins from supragingival plaque were significantly different from proteins on the tongue. It is worth noting that the buccal mucosa, supragingival plaque and the tongue are the three oral sites with the largest numbers of samples (see **Table 1** and **Figure 1B**), suggesting that more intense sampling of some of the other minor sites may uncover fine-scale differences in carbon content as well. For analysis of minor body sites in the remainder of the paper, we restrict our discussion to the three oral sites with sufficient sampling to obtain statistical significance, noting that further exploration of differences across other body sites would be an interesting extension of the current work.

As has been documented in other studies (Baudouin-Cornu et al., 2004; Bragg and Hyder, 2004), there was a negative relationship between the carbon content of protein sequences, and the GC content of the corresponding coding regions (**Figure 1C**). Nevertheless, the effect of major body site remained significant (ANCOVA; Type I ss; *F* = 273.3, *df* = 4, *p <* 2 × 10^-16^) when we included GC content as a covariate (ANCOVA; *F* = 1719.4, *df* = 1, *p <* 2 × 10^-16^). This suggests that selection forces driving variation in GC content across body sites cannot fully explain differences in protein carbon fractions. Similar to findings from large-scale ecological systems, we found that it was not only the median, but also the variance in C content that changed from one body site to another (see Appendix II, Table A.2.3, and Figure A.2.1). Skin and nasal sites, for example, had significantly larger variances in C content as compared to the other three body sites, with oral and vaginal sites having intermediate variances and stool having the smallest variance (though not significantly different from the variance of vaginal sites).

### Oxygen Content

**FIGURE 2:**
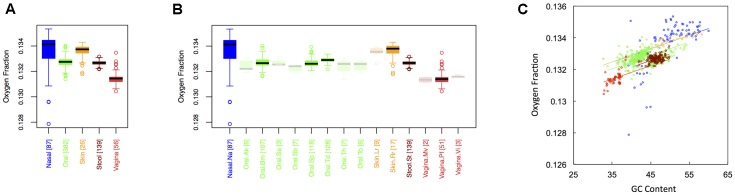
Boxplots showing the average oxygen content of microbial proteins as a function of human body site for **(A)** the 5 major sites and **(B)** the 15 minor sites in our study. The number of samples from each site is indicated in square brackets on axis labels. Faded boxes are used for sites with fewer than 10 samples. Differences in oxygen content were tested with Dunn’s test. Statistics are summarized in Appendix II, Tables A.2.4, A.2.5; **(C)** Scatterplot of oxygen content versus GC content with associated regression lines from our ANCOVA analysis for major body sites: nasal (blue), oral (green), skin (yellow), stool (brown), and vaginal (red).

In **Figure 2**, we show boxplots of the average oxygen fraction of microbial proteins from major (**Figure 1A**) and minor (**Figure 1B**) body sites. As was the case with carbon, nasal and skin samples were not significantly different from one another. All other body site comparisons, however, indicated broad scale variation in oxygen use (see Appendix II, Table A.2.4). Nasal and skin microbiomes had the highest oxygen content, followed by oral, stool and, finally, vaginal microbiomes (O_nasal_ ∼ O_skin_ > O_oral_ > O_stool_ > O_vaginal_). Similar to carbon content, oxygen content at minor sites was largely predictable based on major site differences (see Appendix II, Table A.2.5). Once again, however, there were fine-scale trends apparent among oral sites. For example, the oxygen content the buccal mucosa differed from both supragingival plaque and the tongue, while the oxygen content of the tongue differed from supragingival plaque.

Overall, we found a positive relationship between the oxygen content of protein sequences, and the GC content of the corresponding coding regions (**Figure 2C**). As with carbon, however, the effect of major body site remained significant (ANCOVA; Type I ss; *F* = 43.2, *df* = 4, *p <* 2 × 10^-16^) when we included GC content as a covariate (ANCOVA; *F* = 738.4, *df* = 1, *p <* 2 × 10^-16^). Also similar to carbon, oxygen content showed differences in variance among body sites (see Appendix II, Table A.2.6, and Figure A.2.2), being largest at nasal sites and smallest in stool.

### Nitrogen Content

**FIGURE 3:**
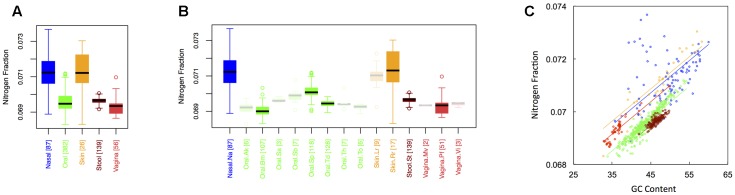
Boxplots showing the average nitrogen content of microbial proteins as a function of human body site for **(A)** the 5 major sites and **(B)** the 15 minor sites in our study. The number of samples from each site is indicated in square brackets on axis labels. Faded boxes are used for sites with fewer than 10 samples. Differences in nitrogen content were tested with Dunn’s test. Statistics are summarized in Appendix II, Tables A.2.7, A.2.8; **(C)** Scatterplot of nitrogen content versus GC content with associated regression lines from our ANCOVA analysis for major body sites: nasal (blue), oral (green), skin (yellow), stool (brown), and vaginal (red).

Average nitrogen fraction of microbial proteins from major (**Figure 1A**) and minor (**Figure 1B**) body sites is shown in **Figure 3**. Comparing **Figures 2**, **3** indicates that, at least for major body sites, trends observed for nitrogen were quite similar to those observed for oxygen. That is, differences between skin and nasal sites were non-significant, with both the nares and skin showing elevated nitrogen as compared to the other three body locations. All other body site comparisons indicated significant differences (see Appendix II, Table A.2.7), with vaginal sites being particularly nitrogen poor (N_nasal_ ∼ N_skin_ > N_stool_ > N_oral_ > N_vaginal_). For minor body sites (see Appendix II, Table A.2.8), differences in the nitrogen content of proteins once again appeared between the buccal mucosa, supragingival plaque and the tongue. Unexpectedly, though, whereas major body sites that were relatively high in oxygen were also high in nitrogen, the same was not true for minor body sites. Within the oral microbiome, for example, supragingival plaques exhibited relatively low oxygen content. By contrast, this was one of the most nitrogen-rich sites in the mouth. At the same time, the buccal mucosa, which was relatively oxygen-rich, was the most nitrogen poor. Thus, whereas oxygen and nitrogen content appear to exhibit broad scale similarities across major body sites, these similarities seem to break down at finer scales of resolution.

Like carbon, the nitrogen content of proteins is known to depend on the GC content of coding regions (Bragg and Hyder, 2004). Specifically, nitrogen content increases with increasing GC content – a trend that we also observed (**Figure 3C**). However, once again, we found that there was still an effect of major body site (ANCOVA; Type I ss; *F* = 218.64, *df* = 4, *p <* 2 × 10^-16^) when we included GC content as a covariate (ANCOVA, *F* = 1901.1, *df* = 1, *p <* 2 × 10^-16^). Differences in the variability of nitrogen content among body sites were also apparent (see Appendix II, Table A.2.9, and Figure A.2.3). As before, nasal and skin sites were not significantly different, and exhibited the largest variances of any of the five body sites. Likewise, the variance of the nitrogen content of stool was significantly smaller than any of the other body sites.

### Sulfur Content

**FIGURE 4:**
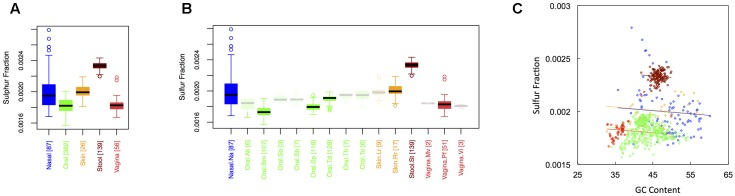
Boxplots showing the average sulfur content of microbial proteins as a function of human body site for **(A)** the 5 major sites and **(B)** the 15 minor sites in our study. The number of samples from each site is indicated in square brackets on axis labels. Faded boxes are used for sites with fewer than 10 samples. Differences in sulfur content were tested with Dunn’s test. Statistics are summarized in Appendix II, Tables A.2.10, A.2.11; **(C)** Scatterplot of sulfur content versus GC content with associated regression lines from our ANCOVA analysis for major body sites: nasal (blue), oral (green), skin (yellow), stool (brown), and vaginal (red).

**Figure 4** shows boxplots of the average sulfur fraction of microbial proteins from major (**Figure 1A**) and minor (**Figure 1B**) body sites. With respect to sulfur, stool had the highest fraction, followed by skin and nasal regions, and then by oral and vaginal sites (S_stool_ > S_skin_ ∼ S_nasal_ > S_oral_ ∼ S_vaginal_). For minor sites, we again saw the buccal mucosa differing from supragingival plaque and the tongue, while supragingival plaque differed from the tongue. Interestingly, sulfur was lowest in the buccal mucosa, but was particularly high in the tongue. Again, ANCOVA indicated an effect of major body site (ANCOVA; Type I ss; *F* = 511.7, *df* = 4, *p <* 2 × 10^-16^), even after accounting for the influence of GC content (ANCOVA, *F* = 221.6, *df* = 1, *p <* 2 × 10^-16^). In terms of variance, nasal and skin sites exhibited the highest variation, while vaginal and stool sites exhibited the lowest (see Appendix II, Table A.2.12, and Figure A.2.4).

### C:N Ratio

**FIGURE 5:**
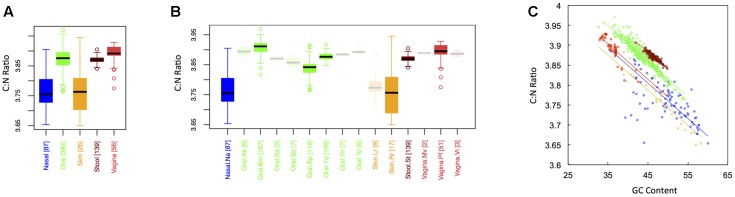
Boxplots showing the average C:N ratio of microbial proteins as a function of human body site for **(A)** the 5 major sites and **(B)** the 15 minor sites in our study. The number of samples from each site is indicated in square brackets on axis labels. Faded boxes are used for sites with fewer than 10 samples. Differences in C:N ratio were tested with Dunn’s test. Statistics are summarized in Appendix II, Tables A.2.13, A.2.14; **(C)** Scatterplot of C:N ratio versus GC content with associated regression lines from our ANCOVA analysis for major body sites: nasal (blue), oral (green), skin (yellow), stool (brown), and vaginal (red).

In keeping with the historical importance of the C:N ratio in ecological stoichiometry, we considered the C:N ratio of microbial proteins across the human body. This is shown in **Figure 5** for both major (**Figure 1A**) and minor (**Figure 1B**) body sites. Like the carbon fraction, C:N ratios were lowest at skin and nasal sites, which were not significantly different from one another. Interestingly, we found that stool and oral microbiomes exhibited intermediate C:N ratios (which were not significantly different), while the vaginal microbiome exhibited the highest (CN_vaginal_ > CN_oral_ ∼ CN_stool_ > CN_nasal_ ∼ CN_skin_). This is in contrast to carbon content, where stool was highest. Notice, however, that the vagina was extremely nitrogen poor, explaining its inflated C:N ratio. In other words, the high C:N ratio in the vagina is driven by both high carbon content and low nitrogen content of vaginal proteins. Again, major body site remained an important determinant of sample C:N ratio (ANCOVA; Type I ss; *F* = 405.82, *df* = 4, *p <* 2 × 10^-16^), even after accounting for dependence on GC content (ANCOVA; *F* = 3878.3, *df* = 1, *p <* 2 × 10^-16^). Similar to both C content and N content, C:N ratios exhibited the greatest variance at skin and nasal sites, intermediate variance at oral and vaginal sites, and the smallest variance in stool (see Appendix II, Table A.2.15, and Figure A.2.5).

### Amino Acid Compositions

**FIGURE 6:**
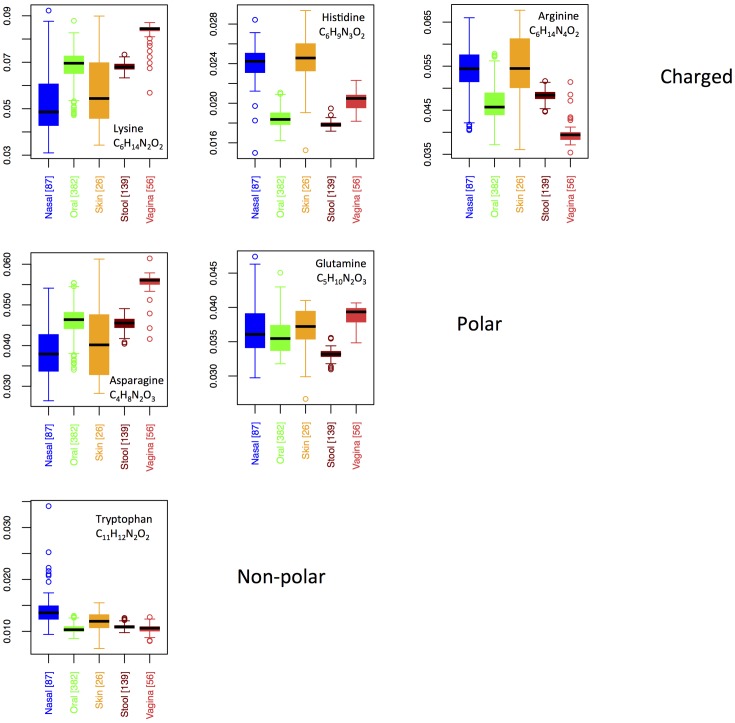
Boxplots showing the fractions of specific N-rich amino acids as a function of body site. Amino acids are grouped according to their chemical properties.

Six AAs have nitrogen in their side chain. **Figure 6** shows boxplots for site-specific usage of these AAs. From **Figure 6**, it is clear that the overabundance of nitrogen in proteins from the skin and nares is primarily a result of higher usage of arginine and histidine at these locations. While both arginine and histidine are positively charged and basic, it does not appear that this feature is driving differences among body sites. Indeed, lysine, which is also positively charged and basic is actually utilized significantly less on skin and in the nares. Moreover, because the pKa of lysine falls between that of histidine and arginine, it suggests that acidity of the protein residues is also not the primary factor under selection. Notably, however, lysine provides the chemical properties of a positively charged, basic AA with only two nitrogen atoms, as opposed to the three found in histidine and the four found in arginine. Thus, organisms trying to conserve nitrogen may favor lysine in place of histidine or arginine, whereas organisms living in environments where nitrogen is not limiting are likely to use histidine and arginine freely. This supports our hypothesis that nutrient limitation, rather than specific structural or chemical requirements, may be driving body site differences in protein elemental composition.

**FIGURE 7:**
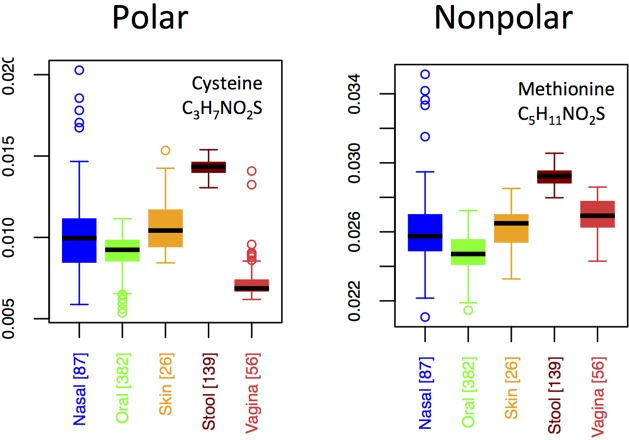
Boxplots showing the fractions of the two S-rich amino acids as a function of body site.

By contrast to the variable usage of different nitrogen-rich AAs across body sites, both of the sulfur-rich AAs are incorporated in qualitatively similar patterns, except that methionine exhibits a relatively higher usage than cysteine in the vagina (**Figure 7**). Although both cysteine and methionine play roles in protein stability, cysteine is polar and forms disulfide bridges, whereas methionine is non-polar and generally non-reactive. Thus, if functionality alone were driving selective incorporation of sulfur-rich AAs in the gut, one might expect differences in the usages of cysteine and methionine in the gut relative to other body sites. Because we do not see this (except in vaginal samples), it suggests that elemental availability, rather than chemical function or specific structural requirements, may be responsible for body site differences. On the other hand, the differing usage patterns of cysteine relative to methionine in the vagina hint that function, rather than nutrient availability, may be driving usage patterns at this site.

**FIGURE 8:**
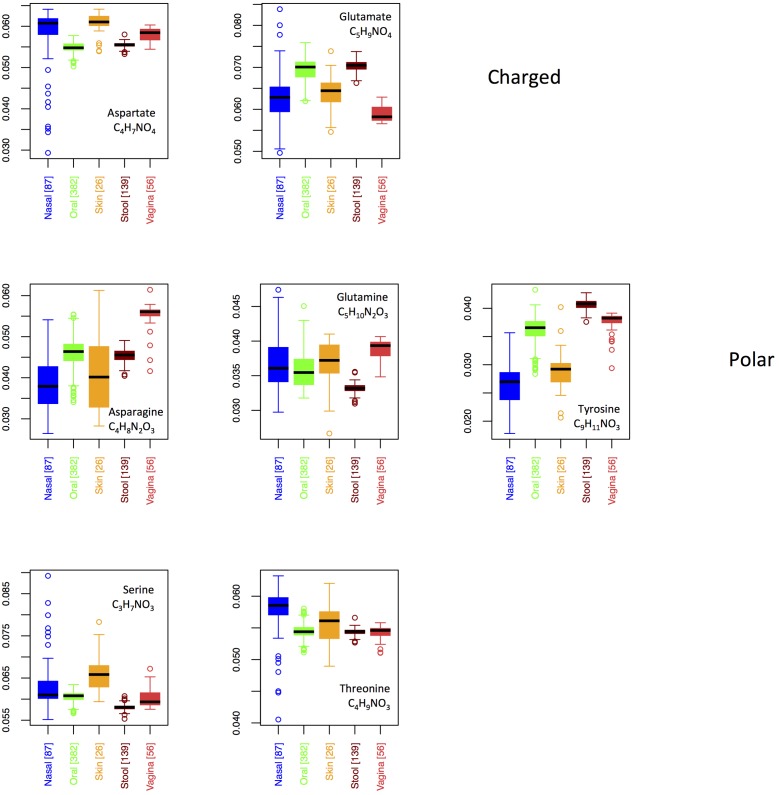
Boxplots showing the fractions of the O-rich amino acids as a function of body site. Amino acids are grouped according to their chemical properties.

In **Figure 8**, we show boxplots for site-specific usage of the seven oxygen-rich AAs. From these, it appears that the low oxygen content of vaginal proteins is largely a result of reduced use of glutamate, whereas the increased oxygen content of the skin and nares seems to stem from increased use of aspartate and serine, with threonine also being enriched in the nares. Notably, glutamate provides an acidic side-chain for a higher C:O ratio than aspartate, thus the patterns that we observe could be explained by glutamate and aspartate substituting for one another in oxygen-poor and oxygen-rich environments, respectively. By contrast, if functionality was driving selection, one would expect both aspartate and glutamate to be over-represented at the same sites. This argument, however, is weakened by the observed patterns in asparagine and glutamine. In particular, both asparagine and glutamine provide a carboxamide functionality, with glutamine exhibiting the higher C:O ratio. Thus, if oxygen availability was truly driving selective incorporation of specific oxygen-rich AAs, we would expect to see similar patterns in glutamate and glutamine and in aspartate and asparagine. In fact the opposite is true. Consequently, while functionality alone does not appear to govern body site differences in oxygen-rich AA incorporation, it is not clear that this is driven by oxygen availability either.

## Discussion

With the recent expansion of human microbiome research, scientists are rapidly acquiring more and more information about the identities of the species living on the human body. This information has important relevance for human health and disease (Cho and Blaser, 2012). However, the biological factors and mechanisms governing microbial community composition, both at different human body sites and across different people, remain largely unknown. Lacking such insight, it is difficult to understand why specific organisms inhabit specific body niches, how this breaks down in the context of dysbiosis, and how dysbiosis might be anticipated, or even prevented, to support human health and well-being.

One framework for interpreting ecosystem function in macroscopic communities is ecological stoichiometry (Sterner and Elser, 2002). Reductionist in nature, ecological stoichiometry seeks to explain community structure, including species interactions, species life histories and even selection for one species over another, within the context of chemical requirements and constraints. By applying ecological stoichiometry to the human microbiome, the goal is to begin to address the chemical underpinnings of microbiome function. This is important, because it takes the focus off of taxonomic/biological mechanisms and moves it to chemical mechanisms. Whereas biological mechanisms are complex, and often compounded by sophisticated or incompletely characterized biological behaviors, chemical mechanisms are more straightforward, primarily limited by stoichiometric and energetic constraints. Further, whereas biology is frequently typified by redundancy, chemistry, at least at an elemental level, is not. Because elements cannot substitute for one another without directly altering mechanism/function, it is often easier to detect underlying patterns from chemical versus biological data. Finally, a chemical approach provides a more straightforward path to therapeutic strategies. In particular, determining how to remove certain problematic organisms, or cultivate beneficial ones requires an understanding of community assembly and the various species interactions involved in community organization. Indeed, simply ‘adding’ a beneficial organism is likely to fail, because that organism will usually be out-competed by the rest of the community. By contrast, determining how to alter the underlying chemical constraints of a system may be as simple as eating specific foods with the desired chemical ratios (gut microbiome) or applying certain nutrient-rich creams to the skin or vagina.

Interestingly, we find that different body sites harbor microbes with distinctly different protein compositions. Skin and nasal sites, for example, are characterized by proteins with high nitrogen and oxygen content. By contrast, carbon and sulfur contents are highest in proteins from stool. Surprisingly, vaginal sites are relatively nutrient-poor, having low nitrogen, oxygen, and sulfur content, and relatively high carbon content (though less than stool). Oral sites tend to be intermediate in carbon, nitrogen, oxygen, and sulfur. The differences that we observe in protein elemental content across body sites provide evidence that the microbiomes at these sites may be differentially limited by nutrient supply rates. However, additional factors beyond nutrient supply are known to impact protein composition. Most obvious is the relationship between protein elemental content and the GC content of CDSs. Because of this relationship, body site differences in protein composition may be generated indirectly as a result of differing selective forces acting on the nucleotide sequence, rather than the protein sequence. The selective forces acting on the nucleotide sequence might have little to do with nutrient supply rates. For example, high GC content appears to be favored in organisms exposed to UV radiation (Mcewan et al., 1998) – something that is likely at skin sites. Consequently, stoichioproteomic trends at skin sites may be driven by UV exposure, rather than nutrient supply rates. In particular, UV radiation may favor higher GC content on the skin, which then automatically translates into the lower carbon content, and higher oxygen and nitrogen contents that we observe at this site. Although this is a compelling mechanistic argument, our analysis shows that GC content alone is insufficient to explain protein elemental variation across body sites. Indeed, while all elements show the expected trends as a function of GC content of their CDSs, there is additional body site variation not explained by GC content. This suggests that selective forces acting on factors other than the nucleotide sequence are at least partially responsible for the protein elemental trends that we observe across different body sites. Further, while we cannot fully rule out structural or chemical requirements as the source of this selection, differing patterns of use of AAs with similar chemical properties across different body sites provides preliminary evidence against the chemical/structural argument and for the argument of differential nutrient supplies.

In addition to the absolute elemental content of proteins at different body sites, another interesting property is variation in protein composition. By and large, the most variation is observed at skin and nasal sites. The least variation is observed in stool. To some extent, this is not surprising, and likely derives in part from relative differences in CDS numbers across sites (see Appendix III, Figure A.3.1). In particular, stool has the largest number of CDSs per sample (median 183712), whereas the nares has the least (median 2188). As a consequence, estimates of sample elemental content at stool sites will generally include a larger number of data points as compared to nasal sites, tightening the precision of stool estimates relative to nasal estimates. Nevertheless, trends in variation are not fully explained by CDS numbers. Skin sites, for example, have approximately 10× as many CDSs as nasal sites, but exhibit similar levels of protein elemental variation. Meanwhile, vaginal sites have CDS counts similar to nasal sites (median 2907), but exhibit much lower variation in elemental content. Indeed, the elemental variation at vaginal sites is often on par with the variation at oral sites, which have much larger numbers of CDS per sample (median 129778). Larger variation in elemental content at a site might indicate larger habitat heterogeneity. For example, the larger variation in nasal versus vaginal sites might indicate a greater degree of variation in nutrient supply rates (or other selective factors) in the nose relative to the vagina. Because much of this variation comes from measures across individuals, this habitat heterogeneity is likely a result of greater inter-individual variation at these sites.

While many studies in ecological stoichiometry seek to define differences in the elemental compositions of organisms across habitats (Hecky et al., 1993; Elser and Hassett, 1994; Cardinale et al., 1997; Elser et al., 2000a; Sterner and Elser, 2002), a broader goal is to use this information to try to understand ecosystem function. With this in mind, we briefly discuss the various different habitats that we considered in our study, attempting to interpret our findings within the context of the specific environmental conditions present at these sites.

### Skin and Nasal Sites

Skin and nasal sites are remarkably similar in all stoichiometric aspects studied here. Indeed, the only significant differences between these two sites were that skin tended to have proteins with a more tightly constrained oxygen content and a more tightly constrained sulfur content (possibly due to less interpersonal variation in oxygen and sulfur supply rates, see above). Perhaps it should not be surprising that the skin and nares are alike. After all, both locations are lined with a similar type of human cell – i.e., stratified squamous keratinizing epithelium – suggesting that the ‘detritus’ supplied to both locations may be similar, at least from the host. Nevertheless, nasal and skin sites (including the retroauricular crease) are known to differ in their microbiome communities (Grice et al., 2009). Most notably, the nares is a common reservoir for *Staphylococcus aureus* which, though also present on exposed skin, is less abundant (Kluytmans et al., 1997). Thus, despite their similarities in host interface, skin and nasal sites still exhibit microbiome differences. For this reason, it is somewhat unexpected that their underlying elemental chemistries are so similar. While we cannot rule out the effects of chemical constraints other than those studied here (e.g., the presence of more complex chemicals like sebum), our preliminary findings suggest that factors other than nutrient availability may be dictating taxonomic differences in nasal versus skin colonization.

One observation that is hard to avoid is that both skin and nasal samples – the two air-exposed sites – are rich in nitrogen and oxygen. It is likely that oxygen limitation at these locations is minimized as a result of their interface with the atmosphere. Indeed, this is in keeping with our hypothesis that body sites associated with large aerobic niches should harbor microbial biomass that is richer in oxygen. While it is also conceivable that nitrogen limitation is minimized as a result of contact with nitrogen in the atmosphere, the high energetic costs of nitrogen fixation make this less probable. Thus, while species like *Klebsiella pneumoniae –* commonly found in the anterior nares (Wilson and Hamilos, 2014) – are known to have nitrogen fixing capabilities (Dixon et al., 1980), it is more likely that the high nitrogen content in skin microbiomes is supplied as nitrates, ammonia, and urea through human sweat (Gilchrist and Benjamin, 2011; Scharschmidt and Fischbach, 2013) or as other nitrogen-rich proteins common on epithelial surfaces.

### Stool

The gut microbiome – rich in carbon and sulfur – should primarily receive nutrients through feeding behavior of the human host (Turnbaugh et al., 2009). However, food taken in by the host is not universally transferred to gut bacteria. Instead, the host must absorb a good deal of the nutrients, setting up competition for potentially limiting elements (Bäckhed et al., 2005). With this in mind, it is perhaps not surprising that carbon and sulfur are in excess in stool. As omnivores, humans will generally exhibit stoichiometric mismatch, such that they consume food with a much higher C:N ratio than their own tissue (Denno and Fagan, 2003). Consequently, in the process of fulfilling human dietary requirements, carbon will be recycled to the environment. Within the human gut, this means that there will be an excess of carbon, whereas nitrogen may be limiting. Bacteria that can accommodate a high C:N ratio may be better able to live in symbiosis with their host, and thus may have been selected over years of human-microbe co-evolution. For similar reasons, it is likely that sulfur will also be in excess, and thus available to gut microbes. In particular, adult dietary protein requirements generally exceed adult sulfur requirements, which are almost universally met in Western diets (Shils and Shike, 2006; Masella and Mazza, 2009). Again, this means excess sulfur is left to be recycled back to the environment, including to the symbiotic microbiota living in the human stomach and intestines. Indeed, microbial sulfur metabolism appears to be common and of great importance within the human intestine, potentially regulating health and disease (Carbonero et al., 2012). That said, the high sulfur content of stool could also reflect the fact that anaerobic organisms – which are known to have higher protein sulfur contents than aerobic organism (Bragg et al., 2006) – form the majority of the gut microbiome. If this is true, however, it is not clear why the vagina, another site with a large fraction of anaerobes (Ravel et al., 2011), is one of the more sulfur-poor microbiomes (see **Figure 4A**).

While the high carbon and sulfur content of gut proteins may be easily rationalized, one finding that is less obvious is the observed tight regulation over ecological stoichiometry in the gut in general. This is somewhat surprising because the supply rate of nutrients to gut microbes should depend on human diet – something that is expected to be highly variable across individuals. An interesting and open question is why the protein elemental stoichiometry of the gut is so tightly regulated, while the elemental stoichiometry of other sites – most notably skin and the nares – is not. This may be because absolute bacterial biomass recovered from the skin and nares is low, as are CDS counts, making stochastic effects on protein elemental ratios more prominent. However, it may also be driven by as yet undetermined constraints on gut elemental protein content. Ultimately, finding an answer to this question may help to elucidate both the structuring principles guiding assembly of gut microbiomes, and the symbiotic host–microbe interactions that these microbiomes undertake.

### Vaginal

The protein content of the vaginal microbiome is remarkably nutrient-poor. This is somewhat unexpected, given the presence of rich vaginal secretions containing proteins and immunoglobulins at concentrations of 15–26 μg/mL, as well as nitrogen-rich urea at concentrations of 49 mg/100 mL (Geshnizgani and Onderdonk, 1992). Whether the low nutrient content of vaginal proteins reflects additional constraints of the vaginal environment [e.g., its low pH (Ravel et al., 2011)], whether this is evidence of competing interests with host processes (e.g., the need for certain sulfur compounds during reproduction), or whether this is simply a result of the carbohydrate component of vaginal secretions being high relative to other components is unclear. Nevertheless, understanding nutrient constraints and exchange in this particular microbial niche could prove fruitful. Indeed, dysbiosis is relatively common in the vagina (Huth, 1989), and may be a result of nutrient imbalances that allow certain bacterial taxa to bloom.

### Oral

Microbial proteins from the oral microbiome are intermediate in nutrient content between those of stool and those of the vagina. In particular, the oral microbiome has a similar nitrogen and oxygen content to stool, but a similar sulfur content to the vagina. Notably, the oral region is the only microbiome in the HMP where more than one sub-site was surveyed extensively (see **Table 1**). Interestingly, we do see fine-scale variation across different oral microbiomes for almost all elements considered. In particular, the buccal mucosa often differs from supragingival plaque which, itself, differs from the tongue. Most notably, supragingival plaque has a relatively higher nitrogen content. While salivary nitrate concentrations are quite high (Granli et al., 1989; Pannala et al., 2003), and could serve as nitrogen sources, it is unclear why nitrate should be particularly available in plaque. A more likely hypothesis is that the high nitrogen content in plaque comes from nitric oxide formed by gingival cells (Schreiber et al., 2010). Interestingly, denitrification in these locations has been documented (Schreiber et al., 2010).

## Conclusion and Future Directions

In this paper, we have taken a step toward untangling the complex ecological stoichiometry of the human microbiome. In particular, we have studied microbial stoichiometry as reflected in the protein composition of the various microbial proteins from different body sites. Although this is a promising start, showing significant variation between both major and minor body sites, a more complete picture will only emerge with further study. Indeed, just as microbial stoichiometry in oceans and soils has focused on whole cell analysis, extending the work presented in the current study to molecules beyond proteins could yield exciting results. At the very least, examining phosphorus content of the various human microbiomes could prove insightful.

Intermediate between the stoichioproteomic approach that we have taken here, and the type of whole-cell ecological stoichiometric analysis that has been applied to microbiomes from oceans, lakes, and rivers, an alternate option would be to consider transcriptomics. Though an RNA analysis would still be restricted to proteins, it would better reflect protein expression rates in specific environments. Indeed, just because an organism has DNA coding for a particular nutrient-rich protein does not mean that protein is expressed under conditions where the nutrient is limiting (Gilbert and Fagan, 2011). By applying a stoichioproteomic analysis to transcriptomics datasets, it would be possible to determine more absolute metrics of nutrient use across the human microbiome. Although the types of transcriptomics datasets that this approach requires are currently limited, this is likely to change in the near future.

Another complicating factor that we have not considered in the current work is the possibility that the trends we observe are driven by phylogenetic conservation of traits. Indeed, elemental contents of proteins are known to be similar across related taxa, while different microbiomes are known to be preferentially populated with organisms from certain taxonomic groups. Although there are undoubtedly selective forces determining the phylogenetic relationships among and between microbial constituents from different microbiomes, these forces may act on factors independent of nutrient use and availability. In other words, nutrient composition may be indirectly selected for as a result of selection acting on some other phylogenetically conserved trait that is differentially important for survival at various body site locations. It would be interesting to try to determine the extent to which nutrient constraints are driving phylogenetic differences among body sites and vice versa.

A final avenue of research would be to better define stoichiometric mismatches between nutrient supply rates and bacterial biomass across the different body sites. Although we have suggested largely speculative explanations for observed differences in C, N, O, and S content of proteins, a true understanding of which elements are limiting at which locations, and how this depends on both host biology and the biology of the microbes in the community, is necessary for a mechanistic understanding of nutrient exchange and nutrient cycling in these systems. Although not an easy task, development of such studies could provide valuable information about host–microbe interactions and host influence on microbiome composition. This, in turn, could be useful for devising therapeutic strategies aimed at maintaining healthy microbiomes in order to preserve the beneficial services that they provide. It could also be used to develop understanding of microbiome dysbiosis, which is known to play a role in a number of diseases, including psoriasis and vaginal bacteriosis. Indeed, ecological stoichiometry may be a particularly advantageous framework for understanding the human microbiome because it brings sequencing studies together with a multiple currency approach to understanding ecosystem function. Ultimately, this allows for a rigorous chemical interpretation of microbial processes. In a time when microbiome data is exploding, but methods for making sense of the data lag behind, ecological stoichiometry may be just the tool that we need for taking the next step in human microbiome science.

## Author Contributions

SB, DK, and WF conceived of the idea; BV-P and SB performed all microbiome and bioinformatics analysis necessary to determine the elemental fractions of the proteins at different body sites; KM, BV-P, and SB performed statistical analysis on protein elemental fractions; BV-P, SB, KM, DK, and WF discussed and interpreted results; SB wrote the paper.

## Conflict of Interest Statement

The authors declare that the research was conducted in the absence of any commercial or financial relationships that could be construed as a potential conflict of interest.

## Footnotes

1 ftp://public-ftp.ihmpdcc.org/HMGI/

2 http://hmpdacc.org/HMGI/

3 https://www.ncbi.nlm.nih.gov/pubmed/21304707

## References

[B1] Acquisti C. Kleffe J. Collins S. (2007). Oxygen content of transmembrane proteins over macroevolutionary time scales. *Nature* 445 47–52. 10.1038/nature05450 17183269

[B2] Andersen T. Hessen D. O. (1991). Carbon, nitrogen, and phosphorus content of freshwater zooplankton. *Limnol. Oceanogr.* 36 807–814. 10.4319/lo.1991.36.4.0807

[B3] Bäckhed F. Ley R. E. Sonnenburg J. L. Peterson D. A. Gordon J. I. (2005). Host-bacterial mutualism in the human intestine. *Science* 307 1915–1920. 10.1126/science.1104816 15790844

[B4] Barsdate R. Prentki R. Fenchel T. (1974). Phosphorus cycle of model ecosystems: significance for decomposer food chains and effect of bacterial grazers. *Oikos* 25 239–251. 10.2307/3543942

[B5] Baudouin-Cornu P. Schuerer K. Marlière P. Thomas D. (2004). Intimate evolution of proteins. Proteome atomic content correlates with genome base composition. *J. Biol. Chem.* 279 5421–5428. 10.1074/jbc.M306415200 14645368

[B6] Baudouin-Cornu P. Surdin-Kerjan Y. Marliere P. Thomas D. (2001). Molecular evolution of protein atomic composition. *Science* 293 297–300. 10.1126/science.1061052 11452124

[B7] Belkaid Y. Segre J. A. (2014). Dialogue between skin microbiota and immunity. *Science* 346 954–959. 10.1126/science.1260144 25414304

[B8] Belkaid Y. Tamoutounour S. (2016). The influence of skin microorganisms on cutaneous immunity. *Nat. Rev. Immunol.* 16 353–366. 10.1038/nri.2016.48 27231051

[B9] Bragg J. G. Hyder C. L. (2004). Nitrogen versus carbon use in prokaryotic genomes and proteomes. *Proc. R. Soc. Lond. B Biol. Sci.* 271 S374–S377. 10.1098/rsbl.2004.0193 PMC1810051 15504022

[B10] Bragg J. G. Thomas D. Baudouin-Cornu P. (2006). Variation among species in proteomic sulphur content is related to environmental conditions. *Proc. R. Soc. Lond. B Biol. Sci.* 273 1293–1300. 10.1098/rspb.2005.3441 PMC1560280 16720405

[B11] Bragg J. G. Wagner A. (2009). Protein material costs: single atoms can make an evolutionary difference. *Trends Genet.* 25 5–8. 10.1016/j.tig.2008.10.007 19010565

[B12] Carbonero F. Benefiel A. C. Alizadeh-Ghamsari A. H. Gaskins H. R. (2012). Microbial pathways in colonic sulfur metabolism and links with health and disease. *Front. Physiol.* 3:448 10.3389/fphys.2012.00448 PMC3508456 23226130

[B13] Cardinale B. Stabler L. Elser J. (1997). Ecological stoichiometry of N and P in pelagic ecosystems: comparison of lakes and oceans with emphasis on the zooplankton-phytoplankton interaction. *Limnol. Oceanogr.* 42 648–662. 10.4319/lo.1997.42.4.0648

[B14] Cebrian J. Kingsolver J. G. (1999). Patterns in the fate of production in plant communities. *Am. Nat.* 154 449–468. 10.1086/303244 10523491

[B15] Cho I. Blaser M. J. (2012). The human microbiome: at the interface of health and disease. *Nat. Rev. Genet.* 13 260–270. 10.1038/nrg3182 22411464 PMC3418802

[B16] Chrzanowski T. H. Kyle M. (1996). Ratios of carbon, nitrogen and phosphorus in *Pseudomonas fluorescens* as a model for bacterial element ratios and nutrient regeneration. *Aquat. Microb. Ecol.* 10 115–122. 10.3354/ame010115

[B17] DeMott W. R. Gulati R. D. Siewertsen K. (1998). Effects of phosphorus-deficient diets on the carbon and phosphorus balance of *Daphnia magna*. *Limnol. Oceanogr.* 43 1147–1161. 10.4319/lo.1998.43.6.1147

[B18] Denno R. F. Fagan W. F. (2003). Might nitrogen limitation promote omnivory among carnivorous arthropods? *Ecology* 84 2522–2531. 10.1890/02-0370 21058571

[B19] Dixon R. Eady R. R. Espin G. Hill S. Iaccarino M. Kahn D. (1980). Analysis of regulation of *Klebsiella pneumoniae* nitrogen fixation (nif) gene cluster with gene fusions. *Nature* 286 128–132. 10.1038/286128a0 6995849

[B20] Elser J. Acharya K. Kyle M. Cotner J. Makino W. Markow T. (2003). Growth rate–stoichiometry couplings in diverse biota. *Ecol. Lett.* 6 936–943. 10.1046/j.1461-0248.2003.00518.x

[B21] Elser J. J. Acquisti C. Kumar S. (2011). Stoichiogenomics: the evolutionary ecology of macromolecular elemental composition. *Trends Ecol. Evol.* 26 38–44. 10.1016/j.tree.2010.10.006 21093095 PMC3010507

[B22] Elser J. J. Dobberfuhl D. R. MacKay N. A. Schampel J. H. (1996). Organism size, life history, and N: P stoichiometry toward a unified view of cellular and ecosystem processes. *BioScience* 46 674–684. 10.2307/1312897

[B23] Elser J. J. Fagan W. F. Denno R. F. Dobberfuhl D. R. Folarin A. Huberty A. (2000a). Nutritional constraints in terrestrial and freshwater food webs. *Nature* 408 578–580. 11117743 10.1038/35046058

[B24] Elser J. J. Fagan W. F. Subramanian S. Kumar S. (2006). Signatures of ecological resource availability in the animal and plant proteomes. *Mol. Biol. Evol.* 23 1946–1951. 10.1093/molbev/msl068 16870683

[B25] Elser J. J. Hassett R. P. (1994). A stoichiometric analysis of the zooplankton-phytoplankton interaction in marine and freshwater ecosystems. *Nature* 370 211–213. 10.1038/370211a0

[B26] Elser J. J. O’brien W. Dobberfuhl D. Dowling T. (2000b). The evolution of ecosystem processes: growth rate and elemental stoichiometry of a key herbivore in temperate and arctic habitats. *J. Evol. Biol.* 13 845–853. 10.1046/j.1420-9101.2000.00215.x

[B27] Elser J. J. Sterner R. Gorokhova E. Fagan W. Markow T. Cotner J. (2000c). Biological stoichiometry from genes to ecosystems. *Ecol. Lett.* 3 540–550. 10.1046/j.1461-0248.2000.00185.x

[B28] Fagan W. F. Siemann E. Mitter C. Denno R. F. Huberty A. F. Woods H. A. (2002). Nitrogen in insects: implications for trophic complexity and species diversification. *Am. Nat.* 160 784–802. 10.1086/343879 18707465

[B29] Geshnizgani A. Onderdonk A. B. (1992). Defined medium simulating genital tract secretions for growth of vaginal microflora. *J. Clin. Microbiol.* 30 1323–1326. 1583140 10.1128/jcm.30.5.1323-1326.1992 PMC265277

[B30] Gevers D. Knight R. Petrosino J. F. Huang K. McGuire A. L. Birren B. W. (2012). The Human Microbiome Project: a community resource for the healthy human microbiome. *PLoS Biol.* 10:e1001377 10.1371/journal.pbio.1001377 PMC3419203 22904687

[B31] Gilbert J. D. Fagan W. F. (2011). Contrasting mechanisms of proteomic nitrogen thrift in *Prochlorococcus*. *Mol. Ecol.* 20 92–104. 10.1111/j.1365-294X.2010.04914.x 21091557

[B32] Gilchrist M. Benjamin N. (2011). *From Atmospheric Nitrogen to Bioactive Nitrogen Oxides, Nitrite and Nitrate in Human Health and Disease.* Berlin: Springer, 9–19. 10.1007/978-1-60761-616-0_2

[B33] Goldman J. C. Caron D. A. Dennett M. R. (1987). Regulation of gross growth efficiency and ammonium regeneration in bacteria by substrate C: N ratio. *Limnol. Oceanogr.* 32 1239–1252. 10.4319/lo.1987.32.6.1239

[B34] Granli T. Dahl R. Brodin P. Bøckman O. (1989). Nitrate and nitrite concentrations in human saliva: variations with salivary flow-rate. *Food Chem. Toxicol.* 27 675–680. 10.1016/0278-6915(89)90122-1 2606404

[B35] Grice E. A. Kong H. H. Conlan S. Deming C. B. Davis J. Young A. C. (2009). Topographical and temporal diversity of the human skin microbiome. *Science* 324 1190–1192. 10.1126/science.1171700 19478181 PMC2805064

[B36] Hecky R. Campbell P. Hendzel L. (1993). The stoichiometry of carbon, nitrogen, and phosphorus in particulate matter of lakes and oceans. *Limnol. Oceanogr.* 38 709–724. 10.4319/lo.1993.38.4.0709

[B37] Herbert D. (1961). The chemical composition of micro-organisms as a function of their environment Symp. *Soc. Gen. Microbiol.* 11 391.

[B38] Herbert D. (1976). “Stoichiometric aspects of microbial growth,” in *Continuous Culture 6: Applications and New Fields*, eds Dean A. C. R. Ellwood D. C. Evans C. G. T. Melling J. (Chichester: Ellis Harwood), 1–30.

[B39] Herbert D. Phipps P. Strange R. (1971). Chapter III chemical analysis of microbial cells. *Methods Microbiol.* 5 209–344. 10.1016/S0580-9517(08)70641-X

[B40] Hessen D. Lyche A. (1991). Inter-and intraspecific variations in zooplankton element composition. *Arch. Hydrobiol.* 121 343–353.

[B41] Human Microbiome Project Consortium (2012a). A framework for human microbiome research. *Nature* 486 215–221. 22699610 10.1038/nature11209 PMC3377744

[B42] Human Microbiome Project Consortium (2012b). Structure, function and diversity of the healthy human microbiome. *Nature* 486 207–214. 10.1038/nature11234 22699609 PMC3564958

[B43] Huth E. J. (1989). Style notes: bacterial vaginosis or vaginal bacteriosis? *Ann. Intern. Med.* 111 553–554. 10.7326/0003-4819-111-7-553 2774386

[B44] Kluytmans J. Van Belkum A. Verbrugh H. (1997). Nasal carriage of *Staphylococcus aureus*: epidemiology, underlying mechanisms, and associated risks. *Clin. Microbiol. Rev.* 10 505–520. 9227864 10.1128/cmr.10.3.505 PMC172932

[B45] Lemoine N. P. Giery S. T. Burkepile D. E. (2014). Differing nutritional constraints of consumers across ecosystems. *Oecologia* 174 1367–1376. 10.1007/s00442-013-2860-z 24380968

[B46] Makino W. Cotner J. Sterner R. Elser J. (2003). Are bacteria more like plants or animals? Growth rate and resource dependence of bacterial C: N: P stoichiometry. *Funct. Ecol.* 17 121–130. 10.1046/j.1365-2435.2003.00712.x

[B47] Masella R. Mazza G. (2009). *Glutathione and Sulfur Amino Acids in Human Health and Disease.* Hoboken, NJ: John Wiley & Sons 10.1002/9780470475973

[B48] Mazel D. Marliére P. (1989). Adaptive eradication of methionine and cysteine from cyanobacterial light-harvesting proteins. *Nature* 341 245–248. 10.1038/341245a0 2506452

[B49] Mcewan C. E. Gatherer D. Mcewan N. R. (1998). Nitrogen-fixing aerobic bacteria have higher genomic GC content than non-fixing species within the same genus. *Hereditas* 128 173–178. 10.1111/j.1601-5223.1998.00173.x 9687237

[B50] Moe S. J. Stelzer R. S. Forman M. R. Harpole W. S. Daufresne T. Yoshida T. (2005). Recent advances in ecological stoichiometry: insights for population and community ecology. *Oikos* 109 29–39. 10.1111/j.0030-1299.2005.14056.x

[B51] Musto H. Naya H. Zavala A. Romero H. Alvarez-Valín F. Bernardi G. (2006). Genomic GC level, optimal growth temperature, and genome size in prokaryotes. *Biochem. Biophys. Res. Commun.* 347 1–3. 10.1016/j.bbrc.2006.06.054 16815305

[B52] Nakano S.-I. (1994). Carbon: nitrogen: phosphorus ratios and nutrient regeneration of a heterotrophic flagellate fed on bacteria with different elemental ratios. *Arch. Hydrobiol.* 129 257–271.

[B53] Pannala A. S. Mani A. R. Spencer J. P. Skinner V. Bruckdorfer K. R. Moore K. P. (2003). The effect of dietary nitrate on salivary, plasma, and urinary nitrate metabolism in humans. *Free Radic. Biol. Med.* 34 576–584. 10.1016/S0891-5849(02)01353-9 12614846

[B54] Peterson J. Garges S. Giovanni M. McInnes P. Wang L. Schloss J. A. (2009). The NIH human microbiome project. *Genome Res.* 19 2317–2323. 10.1101/gr.096651.109 19819907 PMC2792171

[B55] Proctor L. M. (2011). The human microbiome project in 2011 and beyond. *Cell Host Microbe* 10 287–291. 10.1016/j.chom.2011.10.001 22018227

[B56] Ravel J. Gajer P. Abdo Z. Schneider G. M. Koenig S. S. McCulle S. L. (2011). Vaginal microbiome of reproductive-age women. *Proc. Natl. Acad. Sci. U.S.A.* 108 4680–4687. 10.1073/pnas.1002611107 20534435 PMC3063603

[B57] Rhee G.-Y. (1978). Effects of N: P atomic ratios and nitrate limitation on algal growth, cell composition, and nitrate uptake. *Limnol. Oceanogr.* 23 10–25. 10.4319/lo.1978.23.1.0010

[B58] Rocha E. P. Danchin A. (2002). Base composition bias might result from competition for metabolic resources. *Trends Genet.* 18 291–294. 10.1016/S0168-9525(02)02690-2 12044357

[B59] Scharschmidt T. C. Fischbach M. A. (2013). What lives on our skin: ecology, genomics and therapeutic opportunities of the skin microbiome. *Drug Discov. Today Dis. Mech.* 10 e83–e89. 10.1016/j.ddmec.2012.12.003 24273587 PMC3833721

[B60] Schreiber F. Stief P. Gieseke A. Heisterkamp I. M. Verstraete W. de Beer D. (2010). Denitrification in human dental plaque. *BMC Biol.* 8:24 10.1186/1741-7007-8-24 PMC2859859 20307293

[B61] Shils M. E. Shike M. (2006). *Modern Nutrition in Health and Disease.* Philadelphia, PA: Lippincott Williams & Wilkins.

[B62] Sterner R. W. Elser J. J. (2002). *Ecological Stoichiometry: The Biology of Elements from Molecules to the Biosphere.* Princeton, NJ: Princeton University Press.

[B63] Tezuka Y. (1990). Bacterial regeneration of ammonium and phosphate as affected by the carbon: nitrogen: phosphorus ratio of organic substrates. *Microb. Ecol.* 19 227–238. 10.1007/BF02017167 24196360

[B64] Turnbaugh P. J. Ley R. E. Hamady M. Fraser-Liggett C. Knight R. Gordon J. I. (2007). The human microbiome project: exploring the microbial part of ourselves in a changing world. *Nature* 449 804–810. 10.1038/nature06244 17943116 PMC3709439

[B65] Turnbaugh P. J. Ridaura V. K. Faith J. J. Rey F. E. Knight R. Gordon J. I. (2009). The effect of diet on the human gut microbiome: a metagenomic analysis in humanized gnotobiotic mice. *Sci. Transl. Med.* 1 ra14–ra16 10.1126/scitranslmed.3000322 PMC2894525 20368178

[B66] Urabe J. (1993). N and P cycling coupled by grazers’ activities: food quality and nutrient release by zooplankton. *Ecology* 74 2337–2350. 10.2307/1939586

[B67] Wilson M. T. Hamilos D. L. (2014). The nasal and sinus microbiome in health and disease. *Curr. Allergy Asthma Rep.* 14:485 10.1007/s11882-014-0485-x 25342392

