# Supplementary material for: A Stoichioproteomic Analysis of Samples from the Human Microbiome Project
Source: Front Microbiol. 2017 Jul 18;8:1119. doi: 10.3389/fmicb.2017.01119 (PMC5513900; doi:10.3389/fmicb.2017.01119)
Supplement: Supplementary file 1 [file Data_Sheet_1.DOCX]

**Appendix I**

Boxplots comparable to Figures 1A-5A from the main text, but for analysis with only the subset of sites sequenced at Washington University Sequencing Center.

**Figure A.1.1** Boxplots showing the average carbon (A), oxygen (B), nitrogen (C), and sulfur (D) content and C:N ratio (E) of microbial proteins as a function of the five major body sites. Results are for the subset of samples sequenced at Washington University Sequencing Center (compare to Figure 1 from the main text, which included all samples from all sequencing centers).

Boxplots comparable to Figures 1A,B-5A,B from the main text, but for analysis with only the side-chain, rather than the full amino acid (side chain + backbone).

**Figure A.1.2** Boxplots showing the average carbon content of microbial proteins as a function of human body site for (A) the five major sites and (B) the fifteen minor sites in our study (compare to Figure 1 from the main text). The number of samples from each site is indicated in square brackets on axis labels. Faded boxes are used for sites with fewer than 10 samples.

**Figure A.1.3** Boxplots showing the average oxygen content of microbial proteins as a function of human body site for (A) the five major sites and (B) the fifteen minor sites in our study (compare to Figure 2 from the main text). The number of samples from each site is indicated in square brackets on axis labels. Faded boxes are used for sites with fewer than 10 samples.

**Figure A.1.4** Boxplots showing the average nitrogen content of microbial proteins as a function of human body site for (A) the five major sites and (B) the fifteen minor sites in our study (compare to Figure 3 from the main text). The number of samples from each site is indicated in square brackets on axis labels. Faded boxes are used for sites with fewer than 10 samples.

**Figure A.1.5** Boxplots showing the average sulfur content of microbial proteins as a function of human body site for (A) the five major sites and (B) the fifteen minor sites in our study (compare to Figure 4 from the main text). The number of samples from each site is indicated in square brackets on axis labels. Faded boxes are used for sites with fewer than 10 samples.

**Figure A.1.6** Boxplots showing the average C:N ratio of microbial proteins as a function of human body site for (A) the five major sites and (B) the fifteen minor sites in our study (compare to Figure 5 from the main text). The number of samples from each site is indicated in square brackets on axis labels. Faded boxes are used for sites with fewer than 10 samples.

**Appendix II**

***Carbon Content***

**Table A.2.1** *z*-test statistics and *p-*values for carbon fraction comparisons across major body

sites; stars indicate significant differences. Dunn’s test was performed for multiple pairwise comparison after a Kruskal-Wallis test. Significant p-values for pairwise comparison were determined using Benjamini-Hochberg adjustment.

|  | **Nasal** | **Oral** | **Skin** | **Stool** |
| --- | --- | --- | --- | --- |
| **Oral** | -8.783814  0.0000* | –  – | –  – | –  – |
| **Skin** | 1.025082  0.1527 | 6.278751 0.0000* | –  – | –  – |
| **Stool** | -13.21415  0.0000* | -7.702602  0.0000* | -9.526591  0.0000* | –  – |
| **Vaginal** | -12.10702  0.0000* | -7.203398  0.0000* | -9.705722  0.0000* | -1.691673  0.0504 |

**Table A.2.2** Significant differences (*) for carbon fraction comparisons across minor body sites

|  | **Nasal** | **Oral** | | | | | | | | **Skin** | | **Stool** | **Vaginal** | |
| --- | --- | --- | --- | --- | --- | --- | --- | --- | --- | --- | --- | --- | --- | --- |
|  | **Na** | **Ak** | **Bm** | **Sa** | **Sb** | **Sp** | **Td** | **Th** | **To** | **Lr** | **Rr** | **St** | **Mv** | **Pf** |
| **Ak** | ***** | – | – | – | – | – | – | – | – | – | – | – | – | – |
| **Bm** | ***** |  | – | – | – | – | – | – | – | – | – | – | – | – |
| **Sa** | ***** |  |  | – | – | – | – | – | – | – | – | – | – | – |
| **Sb** | ***** |  |  |  | – | – | – | – | – | – | – | – | – | – |
| **Sp** | ***** | ***** | ***** |  |  | – | – | – | – | – | – | – | – | – |
| **Td** | ***** |  | ***** |  |  | ***** | – | – | – | – | – | – | – | – |
| **Th** | ***** |  |  |  |  | ***** |  | – | – | – | – | – | – | – |
| **To** | ***** |  |  |  |  | ***** | ***** |  | – | – | – | – | – | – |
| **Lr** |  | ***** | ***** | ***** | ***** |  | ***** | ***** | ***** | – | – | – | – | – |
| **Rr** |  | ***** | ***** | ***** | ***** | ***** | ***** | ***** | ***** |  | – | – | – | – |
| **St** | ***** |  |  |  |  | ***** | ***** |  |  | ***** | ***** | – | – | – |
| **Mv** | ***** |  |  |  |  |  |  |  |  | ***** | ***** |  | – | – |
| **Pf** | ***** |  |  |  |  | ***** | ***** |  |  | ***** | ***** |  |  | – |
| **Vi** | ***** |  |  |  |  | ***** |  |  |  | ***** | ***** |  |  |  |

**Table A.2.3** Levene’s test statistic (for comparing homogeneity of variance across groups) for carbon content (associated *p-*values shown below; stars indicate significance)

|  | **Nasal** | **Oral** | **Skin** | **Stool** |
| --- | --- | --- | --- | --- |
| **Oral** | 134.8277  0.0000* | –  – | –  – | –  – |
| **Skin** | 3.8151  0.0533 | 16.4676  0.0001* | –  – | –  – |
| **Stool** | 100.8623  0.0000* | 7.5592  0.0062* | 29.4770  0.0000* | –  – |
| **Vaginal** | 32.4366  0.0000* | 0.2341  0.6288 | 7.3152  0.0084* | 1.5755  0.2109 |

**Figure A.2.1** Histograms showing the carbon fraction of microbial proteins by body site.

***Oxygen Content***

**Table A2.4** *z*-test statistics and *p-*values for oxygen fraction comparisons across major body

sites; stars indicate significant differences. Dunn’s test was performed for multiple pairwise comparison after a Kruskal-Wallis test. Significant p-values for pairwise comparison were determined using Benjamini-Hochberg adjustment.

|  | **Nasal** | **Oral** | **Skin** | **Stool** |
| --- | --- | --- | --- | --- |
| **Oral** | 7.498569  0.0000* | –  – | –  – | –  – |
| **Skin** | -0.238796  0.4056 | -4.658364  0.0000* | –  – | –  – |
| **Stool** | 7.980258  0.0000* | 2.020700  0.0241* | 5.355499  0.0000* | –  – |
| **Vaginal** | 13.66887  0.0000* | 10.14041  0.0000* | 10.09271  0.0000* | 7.902870  0.0000* |

**Table A2.5** Significant differences (*) for oxygen fraction comparisons across minor body sites

|  | **Nasal** | **Oral** | | | | | | | | **Skin** | | **Stool** | **Vaginal** | |
| --- | --- | --- | --- | --- | --- | --- | --- | --- | --- | --- | --- | --- | --- | --- |
|  | **Na** | **Ak** | **Bm** | **Sa** | **Sb** | **Sp** | **Td** | **Th** | **To** | **Lr** | **Rr** | **St** | **Mv** | **Pf** |
| **Ak** | ***** | – | – | – | – | – | – | – | – | – | – | – | – | – |
| **Bm** | ***** |  | – | – | – | – | – | – | – | – | – | – | – | – |
| **Sa** |  |  |  | – | – | – | – | – | – | – | – | – | – | – |
| **Sb** | ***** |  | ***** |  | – | – | – | – | – | – | – | – | – | – |
| **Sp** | ***** |  |  |  |  | – | – | – | – | – | – | – | – | – |
| **Td** | ***** | ***** | ***** |  | ***** | ***** | – | – | – | – | – | – | – | – |
| **Th** | ***** |  |  |  |  |  | ***** | – | – | – | – | – | – | – |
| **To** | ***** |  |  |  |  |  | ***** |  | – | – | – | – | – | – |
| **Lr** |  | ***** | ***** |  | ***** | ***** |  | ***** | ***** | – | – | – | – | – |
| **Rr** |  | ***** | ***** |  | ***** | ***** |  | ***** | ***** |  | – | – | – | – |
| **St** | ***** |  |  |  | ***** |  | ***** |  |  | ***** | ***** | – | – | – |
| **Mv** | ***** |  |  |  |  |  | ***** |  |  | ***** | ***** |  | – | – |
| **Pf** | ***** |  | ***** |  |  | ***** | ***** | ***** |  | ***** | ***** | ***** |  | – |
| **Vi** | ***** |  | ***** |  |  |  | ***** |  |  | ***** | ***** |  |  |  |

**Table A.2.6** Levene’s test statistic (for comparing homogeneity of variance across groups) for oxygen content (associated *p-*values shown below; stars indicate significance)

|  | **Nasal** | **Oral** | **Skin** | **Stool** |
| --- | --- | --- | --- | --- |
| **Oral** | 213.3282  0.0000* | –  – | –  – | –  – |
| **Skin** | 8.3449  0.0047* | 21.3340  0.3123 | –  – | –  – |
| **Stool** | 132.2383  0.0000* | 46.0032  0.0000* | 69.4282  0.0000* | –  – |
| **Vaginal** | 30.3412  0.0000* | 2.3862  0.1231 | 3.2406  0.0756 | 28.1916  0.0000* |

**Figure A.2.2** Histograms showing the oxygen fraction of microbial proteins by body site.

***Nitrogen Content***

**Table A2.7** *z*-test statistics and *p-*values for nitrogen fraction comparisons across major body

sites; stars indicate significant differences. Dunn’s test was performed for multiple pairwise comparison after a Kruskal-Wallis test. Significant p-values for pairwise comparison were determined using Benjamini-Hochberg adjustment.

|  | **Nasal** | **Oral** | **Skin** | **Stool** |
| --- | --- | --- | --- | --- |
| **Oral** | 13.31326  0.0000* | –  – | –  – | –  – |
| **Skin** | 0.411346  0.3404 | -7.349500  0.0000* | –  – | –  – |
| **Stool** | 8.984084  0.0000* | -3.567300  0.0002* | 5.317669  0.0000* | –  – |
| **Vaginal** | 11.95557  0.0000* | 3.261703  0.0006* | 8.243533  0.0000* | 5.181325  0.0000* |

**Table A.2.8** Significant differences (*) for nitrogen fraction comparisons across minor body sites

|  | **Nasal** | **Oral** | | | | | | | | **Skin** | | **Stool** | **Vaginal** | |
| --- | --- | --- | --- | --- | --- | --- | --- | --- | --- | --- | --- | --- | --- | --- |
|  | **Na** | **Ak** | **Bm** | **Sa** | **Sb** | **Sp** | **Td** | **Th** | **To** | **Lr** | **Rr** | **St** | **Mv** | **Pf** |
| **Ak** | ***** | – | – | – | – | – | – | – | – | – | – | – | – | – |
| **Bm** | ***** |  | – | – | – | – | – | – | – | – | – | – | – | – |
| **Sa** |  |  |  | – | – | – | – | – | – | – | – | – | – | – |
| **Sb** | ***** | ***** | ***** |  | – | – | – | – | – | – | – | – | – | – |
| **Sp** | ***** | ***** | ***** |  |  | – | – | – | – | – | – | – | – | – |
| **Td** | ***** |  | ***** |  |  | ***** | – | – | – | – | – | – | – | – |
| **Th** | ***** |  |  |  |  | ***** |  | – | – | – | – | – | – | – |
| **To** | ***** |  |  |  | ***** | ***** |  |  | – | – | – | – | – | – |
| **Lr** |  | ***** | ***** |  |  |  | ***** | ***** | ***** | – | – | – | – | – |
| **Rr** |  | ***** | ***** |  |  |  | ***** | ***** | ***** |  | – | – | – | – |
| **St** | ***** | ***** | ***** |  |  | ***** | ***** |  | ***** | ***** | ***** | – | – | – |
| **Mv** | ***** |  |  |  |  | ***** |  |  |  | ***** | ***** |  | – | – |
| **Pf** | ***** |  | ***** |  | ***** | ***** |  |  |  | ***** | ***** | ***** |  | – |
| **Vi** | ***** |  |  |  |  | ***** |  |  |  | ***** | ***** |  |  |  |

**Table A.2.9** Levene’s test statistic (for comparing homogeneity of variance across groups) for nitrogen content (associated *p-*values shown below; stars indicate significance)

|  | **Nasal** | **Oral** | **Skin** | **Stool** |
| --- | --- | --- | --- | --- |
| **Oral** | 55.2010  0.0000* | –  – | –  – | –  – |
| **Skin** | 0.2483  0.6193 | 29.6170  0.0000* | –  – | –  – |
| **Stool** | 173.9578  0.0000* | 105.1199  0.0000* | 130.0917  0.0000* | –  – |
| **Vaginal** | 31.2524  0.0000* | 5.4492  0.0200 | 22.5416  0.0000* | 43.6027  0.0000* |

**Figure A.2.3** Histograms showing the nitrogen fraction of microbial proteins by body site.

***Sulfur content***

**Table A.2.10** *z*-test statistics and *p-*values for sulfur fraction comparisons across major body

sites; stars indicate significant differences. Dunn’s test was performed for multiple pairwise comparison after a Kruskal-Wallis test. Significant p-values for pairwise comparison were determined using Benjamini-Hochberg adjustment.

|  | **Nasal** | **Oral** | **Skin** | **Stool** |
| --- | --- | --- | --- | --- |
| **Oral** | 6.694494  0.0000* | –  – | –  – | –  – |
| **Skin** | -1.395317  0.0905 | -5.462449  0.0000* | –  – | –  – |
| **Stool** | -7.774525  0.0000* | -18.75802  0.0000* | -3.514537  0.0003* | –  – |
| **Vaginal** | 4.835870  0.0000* | 0.232205  0.4082 | 4.805238  0.0000* | 11.94946  0.0000* |

**Table A2.11** Significant differences (*) for sulfur fraction comparisons across minor body sites

|  | **Nasal** | **Oral** | | | | | | | | **Skin** | | **Stool** | **Vaginal** | |
| --- | --- | --- | --- | --- | --- | --- | --- | --- | --- | --- | --- | --- | --- | --- |
|  | **Na** | **Ak** | **Bm** | **Sa** | **Sb** | **Sp** | **Td** | **Th** | **To** | **Lr** | **Rr** | **St** | **Mv** | **Pf** |
| **Ak** |  | – | – | – | – | – | – | – | – | – | – | – | – | – |
| **Bm** | ***** |  | – | – | – | – | – | – | – | – | – | – | – | – |
| **Sa** |  |  |  | – | – | – | – | – | – | – | – | – | – | – |
| **Sb** |  |  | ***** |  | – | – | – | – | – | – | – | – | – | – |
| **Sp** | ***** |  | ***** |  |  | – | – | – | – | – | – | – | – | – |
| **Td** |  |  | ***** |  |  | ***** | – | – | – | – | – | – | – | – |
| **Th** |  |  | ***** |  |  | ***** |  | – | – | – | – | – | – | – |
| **To** |  |  | ***** |  |  | ***** |  |  | – | – | – | – | – | – |
| **Lr** |  |  | ***** |  |  | ***** |  |  |  | – | – | – | – | – |
| **Rr** |  |  | ***** |  |  | ***** |  |  |  |  | – | – | – | – |
| **St** | ***** | ***** | ***** |  | ***** | ***** | ***** |  | ***** |  | ***** | – | – | – |
| **Mv** |  |  |  |  |  |  |  |  |  |  |  |  | – | – |
| **Pf** | ***** |  | ***** |  |  |  | ***** |  |  | ***** | ***** | ***** |  | – |
| **Vi** |  |  |  |  |  |  |  |  |  |  |  | ***** |  |  |

**Table A.2.12** Levene’s test statistic (for comparing homogeneity of variance across groups) for sulfur content (associated *p-*values shown below; stars indicate significance)

|  | **Nasal** | **Oral** | **Skin** | **Stool** |
| --- | --- | --- | --- | --- |
| **Oral** | 98.1237  0.0000* | –  – | –  – | –  – |
| **Skin** | 7.6392  0.0067* | 0.5779  0.4476 | –  – | –  – |
| **Stool** | 91.2023  0.0000* | 61.3032  0.0000* | 27.8986  0.0000* | –  – |
| **Vaginal** | 25.0704  0.0000* | 4.4543  0.0354* | 2.2010  0.1419 | 7.6362  0.0063* |

**Figure A.2.4** Histograms showing the sulfur fraction of microbial proteins by body site.

***C:N Ratio***

**Table A.2.13** *z*-test statistics and *p-*values for C:N ratio comparisons across major body

sites; stars indicate significant differences. Dunn’s test was performed for multiple pairwise comparison after a Kruskal-Wallis test. Significant p-values for pairwise comparison were determined using Benjamini-Hochberg adjustment.

|  | **Nasal** | **Oral** | **Skin** | **Stool** |
| --- | --- | --- | --- | --- |
| **Oral** | -12.84991 0.0000* | –  – | –  – | –  – |
| **Skin** | -0.284739  0.3879 | 7.217539  0.0000* | –  – | –  – |
| **Stool** | -9.794785  0.0000* | 1.892776  0.0324 | -5.968785  0.0000* | –  – |
| **Vaginal** | -12.45646  0.0000* | -4.246096  0.0000* | -8.724376  0.0000* | -5.023286  0.0000* |

**Table A2.14** Significant differences (*) for C:N ratio comparisons across minor body sites

|  | **Nasal** | **Oral** | | | | | | | | **Skin** | | **Stool** | **Vaginal** | |
| --- | --- | --- | --- | --- | --- | --- | --- | --- | --- | --- | --- | --- | --- | --- |
|  | **Na** | **Ak** | **Bm** | **Sa** | **Sb** | **Sp** | **Td** | **Th** | **To** | **Lr** | **Rr** | **St** | **Mv** | **Pf** |
| **Ak** | ***** | – | – | – | – | – | – | – | – | – | – | – | – | – |
| **Bm** | ***** |  | – | – | – | – | – | – | – | – | – | – | – | – |
| **Sa** |  |  |  | – | – | – | – | – | – | – | – | – | – | – |
| **Sb** | ***** |  | ***** |  | – | – | – | – | – | – | – | – | – | – |
| **Sp** | ***** | ***** | ***** |  |  | – | – | – | – | – | – | – | – | – |
| **Td** | ***** |  | ***** |  |  | ***** | – | – | – | – | – | – | – | – |
| **Th** | ***** |  |  |  |  | ***** |  | – | – | – | – | – | – | – |
| **To** | ***** |  |  |  | ***** | ***** |  |  | – | – | – | – | – | – |
| **Lr** |  | ***** | ***** |  |  |  | ***** | ***** | ***** | – | – | – | – | – |
| **Rr** |  | ***** | ***** |  |  |  | ***** | ***** | ***** |  | – | – | – | – |
| **St** | ***** |  | ***** |  |  | ***** | ***** |  | ***** | ***** | ***** | – | – | – |
| **Mv** | ***** |  |  |  |  | ***** |  |  |  | ***** | ***** |  | – | – |
| **Pf** | ***** |  |  |  | ***** | ***** | ***** |  |  | ***** | ***** | ***** |  | – |
| **Vi** | ***** |  |  |  |  | ***** |  |  |  | ***** | ***** |  |  |  |

**Table A.2.15** Levene’s test statistic (for comparing homogeneity of variance across groups) for C:N ratio (associated *p-*values shown below; stars indicate significance)

|  | **Nasal** | **Oral** | **Skin** | **Stool** |
| --- | --- | --- | --- | --- |
| **Oral** | 33.3824  0.0000* | –  – | –  – | –  – |
| **Skin** | 1.6607  0.2002 | 30.0674  0.0000* | –  – | –  – |
| **Stool** | 161.0433  0.0000* | 98.2921  0.0000* | 136.4319  0.0000* | –  – |
| **Vaginal** | 24.7079  0.0000* | 5.7012  0.0174 | 22.5242  0.0000* | 31.2558  0.0000* |

**Figure A.2.5** Histograms showing the C:N ratio of microbial proteins by body site.

**Appendix III**

GC content, number of coding sequences, and average coding sequence length across each of the major body sites.

**Figure A.3.1** Boxplots showing the GC content of coding sequences (A), number of coding sequences (B) and number of amino acids per coding sequence (C) for microbial proteins from samples across the five major body sites. The number of samples from each site is indicated in square brackets on axis labels.

**References**

Andersen, T., and D. O. Hessen. 1991. Carbon, nitrogen, and phosphorus content of freshwater zooplankton. Limnology and Oceanography 36:807-814.

Bäckhed, F., R. E. Ley, J. L. Sonnenburg, D. A. Peterson, and J. I. Gordon. 2005. Host-bacterial mutualism in the human intestine. science 307:1915-1920.

Barsdate, R., R. Prentki, and T. Fenchel. 1974. Phosphorus cycle of model ecosystems: significance for decomposer food chains and effect of bacterial grazers. Oikos:239-251.

Baudouin-Cornu, P., Y. Surdin-Kerjan, P. Marliere, and D. Thomas. 2001. Molecular evolution of protein atomic composition. Science 293:297-300.

Cebrian, J., and J. G. Kingsolver. 1999. Patterns in the fate of production in plant communities. The American Naturalist 154:449-468.

Cho, I., and M. J. Blaser. 2012. The human microbiome: at the interface of health and disease. Nature Reviews Genetics 13:260-270.

Chrzanowski, T. H., and M. Kyle. 1996. Ratios of carbon, nitrogen and phosphorus in Pseudomonas fluorescens as a model for bacterial element ratios and nutrient regeneration. Aquatic Microbial Ecology 10:115-122.

Consortium, H. M. P. 2012. A framework for human microbiome research. Nature 486:215-221.

DeMott, W. R., R. D. Gulati, and K. Siewertsen. 1998. Effects of phosphorus‐deficient diets on the carbon and phosphorus balance of Daphnia magna. Limnology and Oceanography 43:1147-1161.

Denno, R. F., and W. F. Fagan. 2003. Might nitrogen limitation promote omnivory among carnivorous arthropods? Ecology 84:2522-2531.

Eiser, J. J., and R. P. Hassett. 1994. A stoichiometric analysis of the zooplankton-phytoplankton interaction in marine and freshwater ecosystems. Nature 370:211-213.

Elser, J., K. Acharya, M. Kyle, J. Cotner, W. Makino, T. Markow, T. Watts et al. 2003. Growth rate–stoichiometry couplings in diverse biota. Ecology Letters 6:936-943.

Elser, J., W. O'brien, D. Dobberfuhl, and T. Dowling. 2000a. The evolution of ecosystem processes: growth rate and elemental stoichiometry of a key herbivore in temperate and arctic habitats. Journal of Evolutionary Biology 13:845-853.

Elser, J., R. Sterner, E. Gorokhova, W. Fagan, T. Markow, J. Cotner, J. Harrison et al. 2000b. Biological stoichiometry from genes to ecosystems. Ecology Letters 3:540-550.

Elser, J. J., C. Acquisti, and S. Kumar. 2011. Stoichiogenomics: the evolutionary ecology of macromolecular elemental composition. Trends in ecology & evolution 26:38-44.

Elser, J. J., D. R. Dobberfuhl, N. A. MacKay, and J. H. Schampel. 1996. Organism size, life history, and N: P stoichiometry toward a unified view of cellular and ecosystem processes. BioScience 46:674-684.

Elser, J. J., W. F. Fagan, R. F. Denno, D. R. Dobberfuhl, A. Folarin, A. Huberty, S. Interlandi et al. 2000c. Nutritional constraints in terrestrial and freshwater food webs. Nature 408:578-580.

Elser, J. J., W. F. Fagan, S. Subramanian, and S. Kumar. 2006. Signatures of ecological resource availability in the animal and plant proteomes. Molecular biology and evolution 23:1946-1951.

Fagan, W. F., E. Siemann, C. Mitter, R. F. Denno, A. F. Huberty, H. A. Woods, and J. J. Elser. 2002. Nitrogen in insects: implications for trophic complexity and species diversification. The American Naturalist 160:784-802.

Geshnizgani, A., and A. B. Onderdonk. 1992. Defined medium simulating genital tract secretions for growth of vaginal microflora. Journal of clinical microbiology 30:1323-1326.

Gevers, D., R. Knight, J. F. Petrosino, K. Huang, A. L. McGuire, B. W. Birren, K. E. Nelson et al. 2012. The Human Microbiome Project: a community resource for the healthy human microbiome. PLoS Biol 10:e1001377.

Gilbert, J. D., and W. F. Fagan. 2011. Contrasting mechanisms of proteomic nitrogen thrift in Prochlorococcus. Molecular ecology 20:92-104.

Gilchrist, M., and N. Benjamin. 2011. From atmospheric nitrogen to bioactive nitrogen oxides, Pages 9-19 Nitrite and nitrate in human health and disease, Springer.

Goldman, J. C., D. A. Caron, and M. R. Dennett. 1987. Regulation of gross growth efficiency and ammonium regeneration in bacteria by substrate C: N ratio. Limnol. Oceanogr 32:1239-1252.

Granli, T., R. Dahl, P. Brodin, and O. Bøckman. 1989. Nitrate and nitrite concentrations in human saliva: variations with salivary flow-rate. Food and chemical toxicology 27:675-680.

Grice, E. A., H. H. Kong, S. Conlan, C. B. Deming, J. Davis, A. C. Young, G. G. Bouffard et al. 2009. Topographical and temporal diversity of the human skin microbiome. science 324:1190-1192.

Hassett, R., B. Cardinale, L. Stabler, and J. Elser. Ecological stoichiometry of N and P in pelagic ecosystems: Comparison of lakes and oceans with emphasis on the zooplankton-phytoplankton interaction.

Hecky, R., P. Campbell, and L. Hendzel. 1993. The stoichiometry of carbon, nitrogen, and phosphorus in particulate matter of lakes and oceans. Limnology and Oceanography 38:709-724.

Herbert, D. 1961, The chemical composition of micro-organisms as a function of their environment Symp. Soc. Gen. Microbiol 11:7.

Herbert, D. 1976. Stoichiometric aspects of microbial growth. Continuous culture 6:1-30.

Herbert, D., P. Phipps, and R. Strange. 1971. Chapter III chemical analysis of microbial cells. Methods in microbiology 5:209-344.

Hessen, D., and A. Lyche. 1991. Inter-and intraspecific variations in zooplankton element composition. Archiv für Hydrobiologie 121:343-353.

Huth, E. J. 1989. Style notes: bacterial vaginosis or vaginal bacteriosis? Annals of internal medicine 111:553-554.

Kluytmans, J., A. Van Belkum, and H. Verbrugh. 1997. Nasal carriage of Staphylococcus aureus: epidemiology, underlying mechanisms, and associated risks. Clinical microbiology reviews 10:505-520.

Lemoine, N. P., S. T. Giery, and D. E. Burkepile. 2014. Differing nutritional constraints of consumers across ecosystems. Oecologia 174:1367-1376.

Makino, W., J. Cotner, R. Sterner, and J. Elser. 2003. Are bacteria more like plants or animals? Growth rate and resource dependence of bacterial C: N: P stoichiometry. Functional Ecology 17:121-130.

Michail, S., M. Durbin, D. Turner, A. M. Griffiths, D. R. Mack, J. Hyams, N. Leleiko et al. 2012. Alterations in the gut microbiome of children with severe ulcerative colitis. Inflammatory bowel diseases 18:1799-1808.

Moe, S. J., R. S. Stelzer, M. R. Forman, W. S. Harpole, T. Daufresne, and T. Yoshida. 2005. Recent advances in ecological stoichiometry: insights for population and community ecology. Oikos 109:29-39.

Nakano, S.-I. 1994. Carbon: nitrogen: phosphorus ratios and nutrient regeneration of a heterotrophic flagellate fed on bacteria with different elemental ratios. Archiv für Hydrobiologie 129:257-271.

Nielsen, S. L., S. Enriquez, C. Duarte, and K. Sand-Jensen. 1996. Scaling maximum growth rates across photosynthetic organisms. Functional Ecology:167-175.

Pannala, A. S., A. R. Mani, J. P. Spencer, V. Skinner, K. R. Bruckdorfer, K. P. Moore, and C. A. Rice-Evans. 2003. The effect of dietary nitrate on salivary, plasma, and urinary nitrate metabolism in humans. Free Radical Biology and Medicine 34:576-584.

Peterson, J., S. Garges, M. Giovanni, P. McInnes, L. Wang, J. A. Schloss, V. Bonazzi et al. 2009. The NIH human microbiome project. Genome research 19:2317-2323.

Proctor, L. M. 2011. The human microbiome project in 2011 and beyond. Cell host & microbe 10:287-291.

Ravel, J., P. Gajer, Z. Abdo, G. M. Schneider, S. S. Koenig, S. L. McCulle, S. Karlebach et al. 2011. Vaginal microbiome of reproductive-age women. Proceedings of the National Academy of Sciences 108:4680-4687.

Rhee, G.-Y. 1978. Effects of N: P atomic ratios and nitrate limitation on algal growth, cell composition, and nitrate uptake. Limnol. Oceanogr 23:10-25.

Schreiber, F., P. Stief, A. Gieseke, I. M. Heisterkamp, W. Verstraete, D. de Beer, and P. Stoodley. 2010. Denitrification in human dental plaque. BMC biology 8:24.

Sears, C. L., and W. S. Garrett. 2014. Microbes, microbiota, and colon cancer. Cell host & microbe 15:317-328.

Sterner, R. W., and J. J. Elser. 2002, Ecological stoichiometry: the biology of elements from molecules to the biosphere, Princeton University Press.

Tezuka, Y. 1990. Bacterial regeneration of ammonium and phosphate as affected by the carbon: nitrogen: phosphorus ratio of organic substrates. Microbial Ecology 19:227-238.

Turnbaugh, P. J., R. E. Ley, M. Hamady, C. Fraser-Liggett, R. Knight, and J. I. Gordon. 2007. The human microbiome project: exploring the microbial part of ourselves in a changing world. Nature 449:804.

Turnbaugh, P. J., V. K. Ridaura, J. J. Faith, F. E. Rey, R. Knight, and J. I. Gordon. 2009. The effect of diet on the human gut microbiome: a metagenomic analysis in humanized gnotobiotic mice. Science translational medicine 1:6ra14-16ra14.

Urabe, J. 1993. N and P cycling coupled by grazers' activities: food quality and nutrient release by zooplankton. Ecology 74:2337-2350.

Zeeuwen, P. L., M. Kleerebezem, H. M. Timmerman, and J. Schalkwijk. 2013. Microbiome and skin diseases. Current opinion in allergy and clinical immunology 13:514-520.
